# Sustained Virologic Response to Peginterferon α-2a and Ribavirin in 335 Patients with Chronic Hepatitis C: A Tertiary Care Center Experience

**DOI:** 10.4103/1319-3767.39619

**Published:** 2008-04

**Authors:** Hamad Al Ashgar, Mohammed Q. Khan, Ahmed Helmy, Khalid Al Swat, Abdullah Al Shehri, Abdalla Al Kalbani, Musthafa Peedikayel, Khalid Al Kahtani, Mohammed Al Quaiz, Mohammed Rezeig, Ingvar Kagevi, Mohammed Al Fadda

**Affiliations:** Section of Gastroenterology, Department of Medicine, King Faisal Specialist Hospital and Research Centre, Riyadh, Saudi Arabia

**Keywords:** Antiviral therapy, hepatitis C genotype 4, predictors of viral response, viral relapse

## Abstract

**Background/Aim:**

This retrospective study assessed the efficacy, safety, and the predictors of sustained viral response (SVR) to a 48-week-course of peginterferon α-2a (Pegasys) and ribavirin combination therapy in 335 consecutive Saudi patients with chronic hepatitis C virus (HCV) infection.

**Materials and Methods:**

Clinical, biochemical, and virological parameters were collected at time 0 (pretreatment) and at 12, 24, 48, and 72 weeks posttreatment. The mean ± SD age was 49.1 ± 13.0 years; 229 (68.4%) were males, mean ± SD body mass index was 27.8 ± 7.4, 85 (25.4%) were diabetic, 25 (7.5%) had renal impairment, 136 (40.6%) had previously received interferon ± ribavirin therapy, and 247 (73.7%) underwent pretreatment liver biopsy. Patients with genotypes 1, 2 or 3, 4 and mixed genotype were 60 (22.15%), 30 (11.0%), 148 (54.4%), and 34 (12.5%), respectively.

**Results:**

Early viral response (≥2-log10 HCV-RNA decline 12 weeks posttreatment) was achieved in 253 (75.3%). Patients who completed 48 weeks of treatment were 292 (87.1%); of these, 121 (75.6%) achieved ETVR, 161 (55.1%) continued to have SVR and 60 (20.5%) had a viral relapse following end-of-treatment response, that is 48.1 and 17.9% of all patients (n = 335), respectively. Nonresponders (NR) were 71 (24.3%) patients and 43 (12.8%) were unable to complete treatment (due to side effects or loss to follow up). Compared to the relapsers, patients with SVR were significantly younger (***P*** = 0.000), nondiabetics (***P*** = 0.015), had higher serum albumin (***P*** = 0.007), had less pretreatment inflammatory grade (***P*** = 0.011), infected with genotypes 2 or 3 (***P*** = 0.014), and treatment-naïve patients (***P*** = 0.001). However, in stepwise multivariate logistic regression analysis, only treatment naiveté and low pretreatment inflammatory score were the independent predictors of SVR (***P*** = 0.005 and ***P*** = 0.018, respectively).

**Conclusion:**

Combination therapy, if tolerated and completed, is effective in treating chronic HCV patients, especially those with no previous interferon therapy and lower pretreatment inflammatory grade.

Chronic hepatitis C virus (HCV) infection affects approximately 300 million people worldwide and currently is the most frequent cause for liver transplantation in the United States and Europe.[1] Natural history studies suggest that up to 20% of chronic HCV patients develop liver cirrhosis after 20 years of infection. Moreover, the incidence of chronic liver failure is expected to increase over the next 10 years as a result of the “silent epidemic” of HCV infection.[2]

The recommended treatment for patients with HCV genotypes 1 and 4 is pegylated interferon plus ribavirin for 48 weeks.[3] Such treatment has yielded overall sustained viral response (SVR) rates of 54–63% in randomized controlled phase III clinical trials.[4–6] However, treatment responses are not uniform across all populations,[7] and are dependent on various viral and host factors. The majority of studies conducted worldwide have assessed the predictors of SVR in patients infected with HCV genotypes 1, 2, and 3, and showed that the factors independently associated with a favorable treatment result include serum HCV-RNA levels below 2 million copies/mL (800,000 IU/mL), body weight <75 kg, age below 40 years, the absence of bridging fibrosis or cirrhosis in pretreatment liver biopsy, virus genotypes 2 or 3, and a favorable initial viral kinetic response.[4–6,8–10]

There are limited reports on HCV genotype 4, the most predominant genotype in the Middle East.[11] Most of the studies reporting on the treatment of chronic HCV from the Middle East (mainly from Saudi Arabia, Egypt, Kuwait, and Qatar) are weakened by inclusion of small numbers of patients, use of conventional interferon, lack of sufficient data on HCV genotype and/or absence of data assessing the predictors of sustained response and/or viral relapse after end-of-treatment response. [12–22] Other studies performed on genotype 4 patients outside the Middle East are subject to the same limitation.[23–25]

Therefore, the primary objectives of this retrospective study conducted among 335 consecutive Saudi patients with chronic HCV infection were to (1) assess the overall efficacy of a 48-week course of peginterferon α-2a (Pegasys) and ribavirin combination therapy, (2) compare between patients who achieved SVR and those who relapsed after end-of-treatment viral response, and (3) define the independent predictors of SVR (persistently undetectable HCV RNA 6 months after cessation of treatment) in these patients.

## MATERIALS AND METHODS

### Patients

A total of 335 consecutive patients with chronic HCV infection referred to King Faisal Specialist Hospital and Research Centre (KFSH and RC), Riyadh, Saudi Arabia, between February 2003 and November 2005 were included in this study. Baseline subject characteristics are shown in Table 1. This study was approved by both the Research Advisory Council and the Research Ethics Committee at KFSH and RC.

**Table 1:** Baseline subject characteristics (n = 335)

Variable	Mean ± SD or n (%)
Age (years)	49.1 ± 13.0
Sex (Male/Female)	229 (68.4)/106 (31.6)
Body mass index (kg/m^2^)	27.8 ± 7.4
Genotype*	
1	60 (22.1)
2 or 3	30 (11.0)
4	148 (54.4)
Other and mixed	34 (12.5)
Diabetes (yes/no)	85 (25.4%)/250 (74.6)
Renal impairment (yes/no)	25 (7.5)/310 (92.5)
Previous interferon therapy (yes/no)	136 (40.6%)/199 (59.4)
Alcohol intake (yes/no)	9 (2.7%)/326 (97.3)
Previous organ transplant† (yes/no)	28 (8.4%)/307 (91.6)
Positive autoantibodies (yes/no)	21 (6.3%)/314 (93.7)
Positive HBV or HIV (yes/no)	76 (22.7%)/259 (77.3)
Liver biopsy	247 (73.7)

SD - Standard deviation; n - Number; HBV - Hepatitis B virus; HIV - Human immunodeficiency virus,

* Genotype was determined in 272 (81.2%) patients,

† Liver, kidney, or bone marrow transplant

The accepted indications for starting treatment were the presence of detectable HCV RNA of any genotype, elevated serum alanine aminotransferase (ALT) >1.5-fold and histologically consistent with chronic hepatitis C using METAVIR scoring system.[26] Patients were not excluded if they were hemophiliacs, had undergone transplantation, were on hemodialysis or had positive serology for hepatitis B or HIV viruses [Table 1]. Patients were excluded if they had overt active autoimmune or thyroid disorders, leukothrombocytopenia, hemolytic anemia, seizure disorders, decompensated cirrhosis, had focal lesion on abdominal ultrasonography or had elevated α-fetoprotein. None of the patients were treated and then excluded from the study. In other words, all those who started the treatment were included in the study analysis.

### Methods

Baseline assessment included clinical history, physical examination, body mass index (BMI), as well as routine hematological, biochemical, serological, and virological tests including that of HCV genotype, HCV polymerase chain reaction (PCR).

After an initial assessment, patients were treated with pegylated interferon (40 kD; Pegasys^®^, F. Hoffmann-La Roche, Basel, Switzerland) at a dose of 180 μg per week plus ribavirin (Copegus^®^, F. Hoffmann-La Roche, Basel, Switzerland) at a dose of 1000–1200 mg daily as per body weight-1000 mg if ≤75 kg and 1200 mg if ≥75 kg-for 48 weeks. Clinical, biochemical, and viral parameters were collected pretreatment and at weeks 12, 24, 48, and 72 of follow up.

Pretreatment liver biopsy for pathological grading and staging was performed in 247 (73.7%) patients. The average period between the time of biopsy and start of treatment was 2.53 months. The hepatic necroinflammatory activity and stage of fibrosis in the biopsies were evaluated according to the METAVIR scoring system.[26]

### HCV RNA assays

Serum HCV RNA was extracted using an automated extraction system. HCV detection and quantification were performed using Abbott Real-Time M2000 RT-PCR assay, utilizing two sets of primers and probes, which target a conserved region of the 5' untranslated region of the genome and an internal control. This assay detects and quantifies HCV genotypes (1–6) with a detection limit that ranges from 30 to 100,000,000 IU/mL, where 1 IU/mL = 4 copies/mL. Prior to treatment, HCV genotype was assayed in 272 (81.2%) patients using INNO-LiPA HCV II (Innogenetics NV, Ghent, Belgium) as previously described.[27]

### Definitions

- The National Institute of Health guidelines state that a drop of ≥2-Log^10^ in serum HCV viral load is indicative of a positive response.
- Early viral response (EVR) was defined as ≥2-log^10^ drop in serum HCV viral load at 12 weeks after start of treatment.
- End-of-treatment viral response (ETVR) was defined as undetectable serum HCV RNA at 48 weeks.
- SVR was defined as persistently undetectable HCV RNA at 72 weeks (6 months after the end of course of treatment).
- Nonresponse (NR) was defined as persistent positive HCV (PCR) after 48 weeks of treatment.

### Data collection and statistical analysis

Data were collected initially using a specialized data collection form, then introduced into a Microsoft Excel worksheet and finally transferred to the statistical package for social sciences version 15.0 (SPSS; Chicago, IL, USA) for analysis. Means of continuous variables were compared using Student's *t*-test, nonparametric tests (Wilcoxon's and Mann-Whitney) or one-way analysis of variance (ANOVA) with Post-Hoc test (Turkey's) as appropriate. The Chi-Square or Fisher's exact tests were used to compare frequencies and proportions. Multivariate stepwise logistic regression analysis was performed to determine the independent predictors of sustained response. A ***P*** value of <0.05 was considered statistically significant. An intention-to-treat analysis was used. Patients who discontinued treatment either due to adverse effects or lost to follow up were not included in the analysis for NR, ETVR, and SVR.

## RESULTS

### HCV genotype

As shown in Table 1, HCV genotype was assayed in 272 patients (82.2%). Of these, 148 (54.4%) had genotype 4, while 60 (22.1%), 18 (6.6%), 12 (4.4%), 4 (1.5%), 2 (0.7%), and 28 (10.3%) had genotypes 1, 2, 3, 5, 6, and mixed genotype, respectively.

### Biochemical response

**Figure 1:**
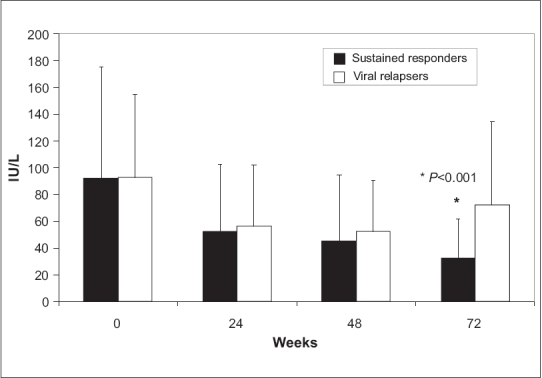
Mean ± SD serum alanine aminotransferase (ALT) levels in both patients with sustained virological response (SVR) and those who relapsed after end-of-treatment response (ETVR) at base line, 24, 48, and 72 weeks

Alanine aminotransferase level at baseline and at weeks 24, 48, and 72 in the sustained responders and those who developed virological relapse are shown in Figure 1. Both groups had similar ALT levels at all times except at week 72, in which those who relapsed after ETVR showed significantly higher ALT levels (***P*** < 0.001).

### Overall viral response

In the entire cohort (***n*** = 335), EVR was achieved in 253 (75.3%). Nonresponse and incomplete treatment (due to side effects or loss to follow up) occurred in 71 (24.3%) and 43 (12.8%) patients, respectively. Patients who completed treatment were 292 (87.1%), of these, 161 (55.1%) achieved SVR and 60 (20.5%) developed virological relapse after end-of-treatment response, that is 48.1% and 17.9% of all patients, respectively [Table 2].

**Table 2:** Viral response rate in relation to the whole cohort and those who completed 48 weeks of treatment

Group	Incomplete therapy	NR	ETVR	SVR	Relapse
Whole cohort (n = 335)	43 (12.8)	71 (21.2)	221 (65.9)	161 (48.1)	60 (17.9)
Completed treatment (n = 292)	-	71 (24.3)	221 (75.6)	161 (55.1)	60 (20.5)

Response rates in patients with genotypes 1 and 4 were similar. Also, they were similar to that seen in the entire cohort (n = 335) and were significantly worse than patients with genotype 2 or 3 (*P* < 0.01). *P* = 0.001 *vs*. pretreated patients, Figures in parentheses are in percentage

### Viral response in relation to previous treatment

**Figure 2:**
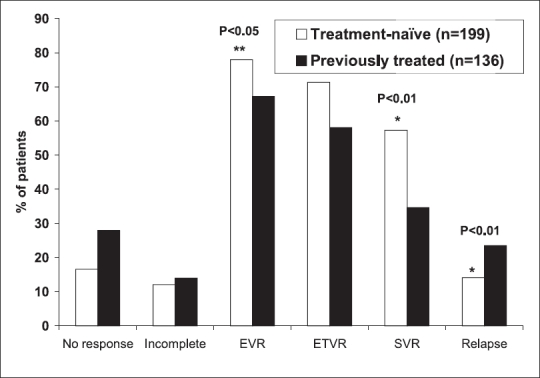
Percentage virological response in treatment naïve patients compared to those who were previously treated with interferon

Viral response rates in the entire cohort, in those who completed treatment and based on whether the patients were previously treated with interferon are shown in Tables 2, 3, and Figure 2. Our results clearly show that the patients who are treatment-naïve have have significantly higher EVR (***P*** = 0.036), higher SVR (***P*** = 0.001), and lower relapse rate after ETVR (***P*** < 0.001).

**Table 3:** Viral response rates in naïve and previously treated patients

Group	Treatment naïve (n = 199)	Previously treated (n = 136)	*P*-value
Incomplete treatment	24 (12.1)	19 (14.0)	NS
Complete treatment	175 (87.9)	117 (86.0)	NS
Nonresponder	33 (18.8)	38 (32.4)	0.034
ETVR	142 (81.1)	79 (67.5)	0.034
Sustained viral responder	114 (65.1)^†^	47 (40.2)	0.001
Relapse after ETVR	28 (16.0)	32 (27.3)	0.001

Figures in parentheses are in percentage, ETVR: end-of-treatment viral response

### Viral response in relation to genotype

**Figure 3:**
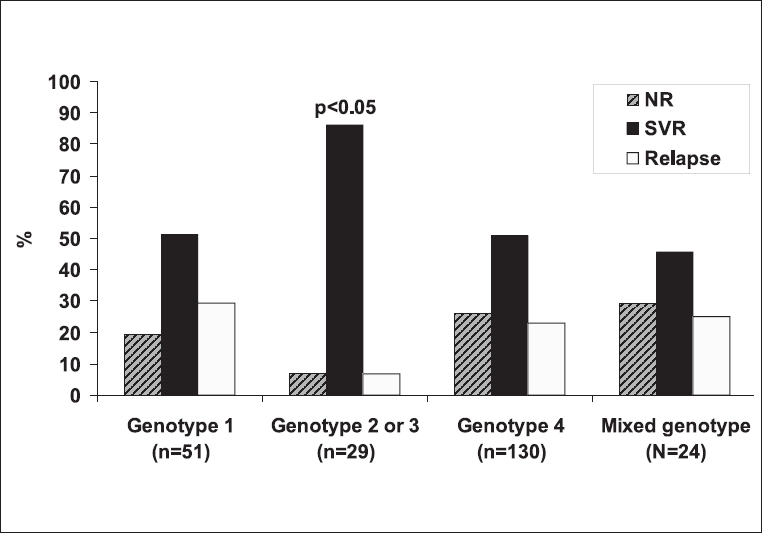
Percentage viral responses in relation to genotype. NR - Nonresponse. SVR - Sustained viral response

To address this issue, we selected the subgroup of patients in whom genotyping was done, having completed 48 weeks of treatment and achieving ETVR. These comprised 234 patients: 51 had genotype 1, 29 had genotype 2 or 3, 130 had genotype 4 and 24 had mixed genotype. Of these, 26 (51.0%), 25 (86.2%), 66 (50.8%), and 11 (45.8%) achieved SVR (***P*** < 0.05 for genotype 2 or 3 vs. either genotype 1 or 4 or the mixed genotype groups) [Figure 3]. However, comparison of SVR between patients with genotypes 1, 4, and those with mixed genotype showed no statistically significant differences (***P*** > 0.05) [Figure 3].

### Viral response in patients with cirrhosis

Only 12 cases of those who had pretreatment liver biopsy had fibrosis stage 4 (cirrhosis). Of these, eight (66.67%) completed 48 weeks of therapy. Three patients were NS and five patients had ETVR. Out of the five patients, only three had SVR and the remaining two relapsed after ETVR.

### Predictors of sustained response

Clinical, laboratory, and pathological characteristics of patients who achieved SVR and those who developed virological relapse after ETVR are shown in Tables 4 and 5. Using the univariate analysis and compared to those who developed viral relapse after ETVR, patients with SVR were significantly younger (***P*** = 0.000), mainly nondiabetics (***P*** = 0.015), had higher levels of serum albumin (***P*** = 0.007), were infected with genotype 2 or 3 (***P*** = 0.014), had less pretreatment necro-inflammatory grade (***P*** = 0.011), and were treatment-naïve (***P*** = 0.001). Both groups were similar regarding pretreatment fibrosis stage and viral load [Table 5]. However, in stepwise multivariate logistic regression analysis (using the abovementioned six variables that were significant in the univariate analysis) only being treatment naive and lower pretreatment necroinflammatory grade were the independent predictors of SVR (***P*** = 0.005 and ***P*** = 0.018, respectively).

**Table 4:** Clinical characteristics of sustained responders versus relapsers after ETVR by univariate analysis

Variable	Unit	Patients with SVR (n = 161)	Relapsers after ETVR (n = 60)	*P* value
Age	years	46.1 ± 13.2	53.2 ± 11.3	0.000
Sex M/F	n (%)	114 (70.8)/47 (29.2)	37 (61.7)/23 (38.3)	NS
Body mass index	kg/m^2^	28.0 ± 7.6	27.3 ± 5.5	NS
Diabetes mellitus	n (%)	27 (16.8)	19 (31.7)	0.015
Renal failure	n (%)	12 (7.5)	3 (5.0)	NS
Previous interferon	n (%)	47 (29.2)	32 (53.3)	0.004
Alcohol intake	n (%)	6 (3.7)	1 (1.7)	NS
Organ transplant	n (%)	13 (8.1)	4 (6.7)	NS
Hemophilia	Yes	12 (7.7)	2 (3.4)	NS
Ribavirin dose	mg/day	923.9 ± 123.3	906.9 ± 155.4	NS
Peginterferon dose	μg/week	176.1 ± 16.3	180.0 ± 0.0	0.062
Ribavirin dose	mg/kg/day	12.8 ± 2.6	12.7 ± 2.5	NS
Peginterferon dose	μg/kg/week	2.5 ± 0.6	2.7 ± 0.7	0.077
EVR*	n (%)	147 (94.8)	52 (88.1)	NS

Data are expressed as mean ± SD or n (%) as appropriate; NS, not significant, i.e, *P* > 0.05. SD - Standard deviation; n - Number; M - Male; F - Female; BMI - Body mass index;

* PCR at 12 weeks posttreatment was performed in 214 patients out of the 221 who completed the treatment, 155 in the SVR group and 59 in the relapsers after ETVR

**Table 5:** Laboratory and pathological characteristics of sustained responders vs. relapsers after ETVR by univariate analysis

Variable	Unit	Patients with SVR (n = 161)	Relapsers after ETVR (n = 60)	*P* value
WBC	×109/L	6.3 ± 2.2	6.0 ± 2.4	NS
Hemoglubin	g/L	143.9 ± 20.2	139.1 ± 18.2	NS
Platelets	×109/L	245.0 ± 94.8	232.9 ± 85.9	NS
INR		0.99 ± 0.13	1.00 ± 0.30	NS
Bilirubin	μmol/L	23.2 ± 125.6	13.8 ± 12.3	NS
ALT	IU/L	92.4 ± 83.5	92.7 ± 62.4	NS
AST	IU/L	65.7 ± 67.3	78.2 ± 53.0	NS
HCV viral load	copy/mL	5.6 ± 8.0 E + 06	7.1 ± 9.5 E + 06	NS
GGT	IU/L	90.4 ± 66.1	122.5 ± 95.9	NS
Albumin	g/L	40.4 ± 4.2	38.7 ± 4.0	0.007
Creatinine	μmol/L	112.7 ± 143.3	99.7 ± 111.5	NS
Cholesterol	mmol/L	3.6 ± 1.4	3.5 ± 1.1	NS
AFP	IU/L	8.1 ± 17.2	11.2 ± 26.2	NS
Genotype 4	n (%)	66 (50.0)*	30 (57.7)†	NS
Genotype 2 or 3	n (%)	18 (19.4)^‡^	2 (6.3)^§^	0.059
Inflammatory grade∥				
0-2	n (%)	103 (88.0)	36 (72.0)	
3-4	n (%)	14 (12.0)	14 (28.0)	0.011
Fibrosis stage∥				
0-2	n (%)	89 (76.1)	35 (70.0)	
3-4	n (%)	28 (23.9)	15 (30.0)	NS

Data are expressed as mean ± SD; NS, not significant, i.e, *P* > 0.05. SD, standard deviation; ALT, alanine aminotransferase; AST, aspartate aminotransferase; ALP, alkaline phosphatase; GGT, γ-glutamyl transferase; AFP, α-fetoprotein.

* Genotyping was done in 132 patients only;

† Genotyping was done in 52 patients only;

∥ Liver biopsy was done in 167 patients out of the 292 who completed the treatment

### Safety profile

A total of 327 side effects were encountered in 170 patients (50.7%) during follow up [Table 6]. The effects that were ≥5% include fatigue, body aches, weight loss, depression, skin rash, anemia, leucopenia, and thrombocytopenia in 29 (8.7%), 17 (5.1%), 30 (9.0%), 20 (6.0%), 20 (6.0%), 63 (18.8%), 73 (21.8%), and 21 (6.3%) patients, respectively. Subcutaneous injections of erythropoietin and granulocyte-colony stimulating factor were used in 17 and 16 patients, respectively. That is, 27 and 21.9% of the patients who developed anemia (***n*** = 63) and leucopenia (***n*** = 73), respectively.

**Table 6:** Frequency of main side effects encountered during therapy

Side effect	Frequency
Fatigue	29 (8.7)
Body aches (myalgia, arthralgia, and headache)	17 (5.1)
Weight loss	30 (9.0)
Itching	15 (4.5)
Skin rash	20 (6.0)
Depression	20 (6.0)
Worsening liver function	3 (0.9)
Thyroid dysfunction*	12 (3.6)
Anemia	63 (18.8)
Leucopenia	73 (21.8)
Thrombocytopenia	21 (6.3)
Fever	8 (3.4)
Others†	13 (3.9)

Data are expressed as n(%);

* Thyroid dysfunction in the form hypo- or hyperthyroidism;

† Others include anorexia (n = 1), nausea (n = 1), cough (n = 5), myopathy (n = 2), neuropathy (n = 2), and nephrosis (n = 1)

## DISCUSSION

This is the biggest cohort of chronic HCV-infected patients treated with the combination of pegylated interferon α-2a and ribavirin to be reported from the Middle East, where infection with genotype 4 is predominant.[11] Our results confirm that patients infected with chronic HCV genotype 4, supported by the work of others,[13,15] can no longer be considered “difficult to treat.” With the use of the 48-week combination regimen and appropriate doses in 335 patients, SVR was achieved in 48.1% with intention-to-treat analysis and in 55.1% of 292 patients who tolerated and completed the full course of therapy. These results are similar to the responses achieved in previous studies that involved cohorts with predominantly HCV genotype 1 infection and less than the patients infected with HCV genotype 2 or 3.[28–30]

The current impression that the patients infected with HCV genotype 4 are difficult to treat and that they respond poorly to interferon therapy was made based on earlier studies where conventional interferon-α was used alone.[12,16,19,20,22,24–26] On the other hand, studies that used pegylated interferon combined with ribavirin on genotype 4 patients showed better results than ours, possibly due to the heterogeneity of our patients and the inclusion of many patients with co-morbidities, associated HBV or HIV infections organ transplant and failed response to previous interferon-based therapy. The SVR was observed in 55.5 and 69% of patients who received treatment for 48 weeks.[13,15] However, these studies have the strength of being prospective and randomized, and only including patients with genotype 4. However, out of a total of 180 and 287 patients, only 40 and 91 patients, respectively, were treated for 48 weeks in these two studies. One study assessed the predictors of SVR.[15] Similarly, SVR was observed in 43.8% of patients after 48 weeks of treatment and only 28 patients had genotype 4.[20] Other studies showed a much lower SVR of 33.3%, a difference that might be related to host- or viral-related factors or to the small number of patients included (only 30 patients).[12]

A total of 71 (24.3%) patients were classified as NR after 48 weeks of therapy and their treatment was discontinued. However, due to the tertiary nature of our hospital and the inclusion of many cases in whom interferon therapy had failed before, cases with organ transplantation and on immunosuppressive therapy, cases co-infected with HIV and/or HBV, cases infected with mixed HCV genotypes and the inclusion of a small number of cases with genotype 2 or 3 (***n*** = 30), this rate of nonresponse is acceptable.

Fibrosis stage by pretreatment liver biopsy was not found to be statistically different between sustained responders and those who developed viral relapse after ETVR. This is contrary to what was previously reported by other studies in both genotypes 1 and 4.[21,31–33] This can be explained by the fact that only 167 out of the 221 patients who completed their course had pretreatment liver biopsy. Of these, only 43 had fibrosis more than stage 2. This means that fibrosis adversely affects the viral response to combination therapy if it exceeds grade 2. In addition, a reason for the lack of significant difference in the fibrosis grades between sustained responders and those who relapsed after ETVR is the time interval between biopsies and the start of therapy.

Baseline HCV viral load was not found to be a predictor of SVR in our study. It is well known that viral load fluctuates and a single reading of HCV quantification may not reflect the actual viral load at the time of treatment, especially if it was assessed at varying intervals from the date of start of treatment. It has also been reported that the differences in interferon response could be secondary to either difference in the viral virulence and/or replication rate among different HCV genotypes and not the absolute viral load.[29]

The safety profile of the combination therapy of pegylated-interferon α-2a and ribavirin used in the present study is comparable to what was previously described in the literature.[34] Indeed, only 43 (12.8%) patients did not complete their course of treatment in our study due to the development of side effects, lost follow-up and/or transfer to liver transplantation or development of contraindication.

The SVR (34.6%) in our patients who previously received interferon therapy is better than that concluded in many studies for both genotypes 1 and 4.[19,21,34] The mechanism(s) underlying this higher response is due to the inclusion of majority of patients who received standard interferon alone (and not combination therapy). The overall low SVR in the previously treated patients may be related to the development of an intrinsic or immunological resistance to the direct antiviral effect of interferon. Mathew ***et al.***[35] reported that the response rates were almost double in patients who were previously treated with interferon monotherapy (24%) compared to those previously treated with combination therapy (12–16%), but without any statistical difference; these values were 28 and 12% in the HALT study.[36] Interestingly, interferon-inducible protein 10 kDa (IP-10), which is a chemokine produced by hepatocytes and targets T-lymphocytes, natural killer cells and monocytes was recently identified.[37,38] Elevated serum levels of IP-10 before initiation of therapeutic intervention for HCV infection were reported in patients not achieving SVR.[39,40] A recent study confirmed that pretreatment IP-10 levels predict SVR in patients infected with HCV genotype 1, even in those with higher BMI and viral load.[41] Thus, assessment of pretreatment IP-10 may help in identifying patients for whom current therapy is beneficial. This protein needs to be tested in patients infected with HCV genotype 4, especially those in whom interferon therapy had failed previously.

The potential limitations of the current study can be summarized in the following points: (1) Posttreatment biopsy was not performed as the main objective of the study was to assess the rate of SVR, which was assayed using serum HCV PCR, relying on the well-established information in the literature that proved stabilization and/or regression in hepatic fibrosis stage in response to treatment, especially if it is associated with viral clearance.[37–39] In addition, assessing the impact of therapy on liver histopathology was beyond the scope of this study. (2) Due to the retrospective nature of the present study, baseline liver biopsy and HCV genotyping were performed in the majority, but not all cases. It is clear that if these two important baseline parameters were performed in all patients, better assessment of their role as predictors of SVR can be done. However, the fact that the percentage of patients who have achieved SVR in the present study is similar to what is already reported in other studies makes it less likely to substantially affect the results.

In conclusion, combination therapy, if tolerated and completed, is effective in treating chronic HCV patients, especially in those with no previous interferon therapy and with low pretreatment inflammatory grade. Persistent efforts and support measures need to be implemented, possibly through a specialized dedicated clinic, to improve patient compliance with follow-up, adherence to therapy, tolerance to treatment, early detection, and subsequent treatment of complications, if any. Further studies addressing the possible underlying mechanism(s) responsible for SVR, including the pretreatment assessment of IP10 levels, are warranted.
